# Management and Outcomes of Hospitalized Patients With Primary Neuroendocrine Tumor and Non-Neuroendocrine Tumor Appendiceal Cancers in the United States

**DOI:** 10.14740/wjon927w

**Published:** 2015-06-12

**Authors:** Olatunji B. Alese, Sungjin Kim, Zhengjia Chen, Walid Shaib, Charles A. Staley, Suresh S. Ramalingam, Taofeek K. Owonikoko, Bassel F. El-Rayes

**Affiliations:** a Department of Hematology and Oncology, Winship Cancer Institute, Emory University, Atlanta, GA, USA; b Samuel Oschin Comprehensive Cancer Institute, Cedars-Sinai Medical Center, Los Angeles, CA, USA; c Department of Biostatistics, Emory University, Atlanta, GA, USA; d Division of Surgical Oncology, Department of Surgery, Winship Cancer Institute, Emory University, Atlanta, GA, USA

**Keywords:** Appendiceal cancer, Surgery, Neuroendocrine, Management outcomes, Hospitalized patients

## Abstract

**Background:**

The incidence of appendiceal cancers continues to rise at a very rapid pace. Although surgery has a central role in the management of appendiceal tumors, literature is lacking regarding the pattern and predictors of surgical treatment for patients with appendiceal cancers. We aimed to describe the surgical treatment for patients with appendiceal cancers, with emphasis on utilization based on histology.

**Methods:**

Hospitalized patients with appendiceal cancer in the US between 2006 and 2010 were included in the study. The Nationwide Inpatient Sample database maintained by the Agency for Health Care Research and Quality was employed for univariate and multivariate testing to identify factors significantly associated with patient outcome.

**Results:**

A total of 3,799 patient discharges were identified over the 5-year period covered by the study. Neuroendocrine tumor (NET) was the diagnosis in 291 (7.66%) patients and non-NET in 3,508 (92.34%) patients. The mean age was 56.8 years (± SD 14.6), with female predominance (54.73% vs. 45.27%). NET patients were younger than those with non-NET (50.7 vs. 57.4 years; P < 0.001). NET patients were more commonly treated with appendectomy compared to non-NET (OR: 1.59; 95% CI: 1.23 - 2.07; P < 0.001). Hyperthermic intraperitoneal chemotherapy (HIPEC) was used in 8.5% of all the cases, mostly in non-NET histology (91% vs. 8%). Majority of the patients treated with HIPEC had no co-morbid medical illness (60%), and received care at high volume hospitals located in urban areas. There was a very low incidence of in-hospital mortality (2.5%).

**Conclusions:**

The described surgical utilization pattern should prompt more research focusing on barriers to appropriate surgical debulking and HIPEC utilization in non-NET appendiceal cancers.

## Introduction

Appendiceal cancers are a heterogeneous group of diseases, with a relatively low incidence compared to other gastrointestinal tumors. Prevalent reports had revealed that appendiceal cancers account for 0.5% of all intestinal tumors [1], with annual incidence of 0.12 cases per 100,000 people [2]. However, a recent study found a remarkable 54% increase in the incidence of appendiceal cancer in the United States over a 10-year period, rising from 0.63 per 100,000 persons in 2000 to 0.97 per 100,000 persons by 2009 [3]. This corresponds to a current estimated 3,000 cases per year in the United States alone. Moreover, survival is strongly associated with stage and histology, which often guide the treatment plan. Since prospective randomized trials are limited in appendiceal tumors, management is largely based on consensus guidelines and institutional series. The largest published series of appendiceal cancers have been the population-based surveillance, epidemiology, and end results (SEER) data reports [2-4]. The most common histologic subtype reported was non-neuroendocrine tumors (non-NETs) which included low grade mucinous (37-38%), adenocarcinoma (26-27%), goblet (15-19%), and signet ring cell carcinoma (4-6%). NETs accounted for 11-17%. Moreover, the impact of histology on survival was highlighted by the wide 5-year disease-specific survival rates for these subtypes ranging from 91% for NET to 27% for signet ring cancers.

Surgical treatment remains the primary management of the majority of appendiceal cancers. Surgical procedures range from simple appendectomy to peritonectomy and intraperitoneal chemotherapy administration. The extent of surgery is influenced by histology, size and stage of the tumor. Given the rarity of these tumors and complexity of their management, there is concern regarding the proper utilization of these surgical approaches [5-7]. The SEER database lacks sufficient details of the management intervention employed for patients entered into the cancer registry database [3]. Consequently, important factors that can impact treatment decisions and outcomes such as patient co-morbidities, surgical expertise as reflected in the characteristics and location of hospital cannot be evaluated using the SEER database. The nationwide inpatient sample (NIS) database is maintained by Agency for Healthcare Research and Quality (AHRQ) and it is the largest database of health care outcome in hospitalized patients in the United States. We utilized this database to assess the pattern and predictors of surgical treatment for patients with appendiceal cancers.

## Methods

The 2006 through 2010 data sets of the NIS were used for this study [8]. The NIS database is the largest all-payer inpatient care database in the United States from over 1,000 hospitals. The database contains clinical and demographic information from hospital discharge abstracts and it is maintained by the Agency for Healthcare Research and Quality (AHRQ). Because the Healthcare Cost and Utilization Project (HCUP) does not release patient identifiers for confidentiality reasons, hospital discharges were used as the unit of measure for analyses. The database codes for diagnosis-related groups (DRGs), procedure and diagnostic indices were used according to the Ninth Edition of the International Classification of Diseases Clinical Modification (ICD-9-CM) [9]. The primary outcome was type of surgery that the patients received. Patient-specific covariates included age, gender, race, histology, insurance status, presence of metastatic disease and co-morbid medical conditions. Histologic diagnosis was divided into NET vs. non-NET cancers which included mucinous, adenocarcinoma, adenosquamous, goblet cell, and signet ring. All instances of hospital discharges for patients with a diagnosis of appendiceal cancer were identified using Clinical Classification Software (CCS) procedure code (231) and ICD-9 Diagnoses (20,911 and 1,535) and procedure codes (9,985, 1,733, 4,573, 9,985, 470; 1 through 9 and 471; 1 through 19) in any fields of principal or secondary diagnosis. Ethical approval was not required for the study since patient information in the database is completely de-identified and the database is legally accessible to the public.

### Statistical analysis

The clinical and demographic characteristics of the patients were summarized using descriptive statistics as appropriate for variable type and distribution. Univariate analysis of surgical treatment with numerical covariates was performed with a logistic model and weighted Chi-square test was used for categorical covariates. Sample stratification, clustering, and weighting were taken into account. Histology, gender, metastatic cancer, co-morbidity, multivisceral resection and HIPEC, location of hospital, number of diagnosis, and stage were found to be significantly related to surgery type. Multivariable analysis of each outcome was further conducted using a backward variable selection method with an alpha level of removal set at 0.1. To simultaneously account for hospital-level and patient-level variation in each endpoint, a generalized linear model with the use of generalized estimated equation was employed. The model accounted for data correlations by assuming exchangeability among admissions from the same hospital. The univariate association of length of stay with type of surgery was evaluated with a negative binomial regression model. Length of stay was first evaluated with a Poisson distribution but due to overdispersion, a negative binomial regression model, which allows for overdispersion, was used for analyses. All analyses were done using SAS 9.3 (SAS Institute, Inc., Cary, NC) with a significant level of 0.05.

## Results

### Characteristics

A total of 3,799 patient discharges with appendiceal cancer identified over the 5-year period covered by the study were included in the analysis (Table 1). There were 3,508 (92.34%) cases of non-NET and 291 (7.66%) were NET. The mean age was 56.8 years (± SD 14.6), with a range of 12 - 97 years. Although there were more female than male patients (54.73% vs. 45.27%), there was no significant difference in histologic distribution by gender (P = 0.172). Metastatic disease was present in 41.3% of the cases. The mean number of concurrent medical diagnoses including the cancer diagnosis was 8 (± 5). The vast majority of the patients (96.8%) had health insurance coverage.

**Table 1:** Descriptive Statistics

Variable	Level	N (%)
Gender	Male (%)	1,716 (45.27)
	Female (%)	2,075 (54.73)
	Missing (%)	8
Race	White (%)	2,319 (61.04)
	Black (%)	286 (7.53)
	Other (%)	1,194 (31.43)
Histology	NET (%)	291 (7.66)
	Non-NET (%)	3,508 (92.34)
Metastatic cancer	Not present (%)	2,228 (58.65)
	Present (%)	1,571 (41.35)
Type of surgery	Appendectomy	698 (18.37)
	Right hemicolectomy	1,356 (35.69)
	Multivisceral resection	324 (8.53)
	HIPEC (%)	91 (2.4)
	Others (%)	483 (12.71)
	Unknown (%)	847 (22.29)
Insurance status	Insured (%)	3,662 (96.78)
	Non-insured (%)	122 (3.22)
	Missing (%)	15
Age (years)	Mean (± SD)	56.86 (± 14.64)
	Median (range)	57 (12 - 97)
Number of diagnoses	Mean (± SD)	8 (± 5)
	Median (range)	8 (1 - 31)
Comorbidity	≥ 1 (%)	1,545 (40.67)
	0 (%)	2,254 (59.33)
Location of hospital	Rural (%)	374 (9.85)
	Urban (%)	3,425 (90.15)
Home discharge	Home (%)	2,792 (73.49)
	Others (%)	1,007 (26.51)
Length of stay	Mean (± SD)	7.56 (± 7)
	Median (range)	5 (1 - 109)

NET: neuroendocrine tumor; HIPEC: hyperthermic intraperitoneal chemotherapy; SD: standard deviation.

A very small proportion of patients with appendiceal cancer (3.2%) in the NIS database were uninsured. The uninsured patients were younger (47 vs. 57 years) and more likely to be African-American (7.5% vs. 2.5%; P = 0.006). There was however no statistically significant difference in health insurance status with respect to gender, co-morbid medical illnesses, or the type of surgical treatment including HIPEC.

### Patient outcome

There was a very low incidence of in-hospital mortality (2.5%) recorded in patients who underwent any type of surgical resections. Patients treated with appendectomy had a lower rate of in-hospital mortality than patients receiving more extensive surgeries. Patients treated with appendectomy had a significantly shorter hospital stay (RR: 0.79; 0.72 - 0.86; P < 0.001) and were more likely to have been discharged home (91% vs. 85%; P < 0.001) rather than to a rehabilitation facility or hospice care.

### Comparison of patient characteristics by tumor histology

Univariate analysis showed significant differences between the NET and non-NET histologic subtypes with respect to age, race, type of surgery, number of diagnosis and presence of metastases (Table 2). Patients with NET were significantly younger (mean age: 50.7 ± 18.02 years) than those with non-NET (57.4 ± 14.21 years; P < 0.001). Metastatic spread of disease was more common in patients with non-NET (95.7% vs. 4.3%; P < 0.001). There was a significant association between NET cancer diagnosis and fewer co-morbid medical conditions (P = 0.012).

**Table 2:** Patient Characteristics and Surgical Intervention in NET and Non-NET Appendiceal Tumors

Covariate	Level	All patients (N = 3,799)	Histology		P-value*
			NET (N = 291)	Non-NET (N = 3,508)	
Age (years)	Mean (± SD)	56.86 (± 14.64)	50.68 (± 18.02)	57.37 (± 14.21)	< 0.001
Gender	Female (%)	2,075 (54.73)	170 (8.19)	1,905 (91.81)	0.172
	Male (%)	1,716 (45.27)	118 (6.88)	1,598 (93.12)	
Race	Black (%)	286 (9.5)	24 (8.39)	262 (91.61)	0.043
	Other (%)	1,194 (31.43)	58 (4.86)	1,136 (95.14)	
	White (%)	2,319 (76.99)	198 (8.54)	2,121 (91.46)	
Type of surgery	Appendectomy (%)	698 (18.37)	91 (13.04)	607 (86.96)	< 0.001
	Right hemicolectomy (%)	1,356 (35.69)	130 (9.59)	1,226 (90.41)	
	Multi-visceral resection (%)	324 (8.53)	27 (8.33)	297 (91.67)	
	HIPEC (%)	91 (2.4)	1 (1.1)	90 (98.9)	
	Others (%)	483 (12.71)	16 (3.31)	467 (96.69)	
	Unknown (%)	847 (22.29)	54 (6.37)	793 (93.62)	
Number of diagnoses	Mean (± SD)	8 (± 5)	7 (± 5)	9 (± 5)	0.012
Metastatic cancer	Not present (%)	2,228 (58.65)	223 (10.01)	2,005 (89.99)	< 0.001
	Present (%)	1,571 (41.35)	68 (4.33)	1,503 (95.67)	
	Relative Risk		0.51 (0.39 - 0.68) P < 0.001	1 (Ref)	

Data are presented as raw number of patients (%) or mean (± SD). *The P-value is calculated by a logistic model for numerical covariates and weighted Chi-square test for categorical covariates. NET: neuroendocrine tumor; HIPEC: hyperthermic intraperitoneal chemotherapy; SD: standard deviation.

### Choice of surgical management approach

Surgical procedures included appendectomy (18.4%), colorectal resections (35.7%), and multivisceral resections (8.5%) (Table 2). Only 2.4% of all the patients had the addition of hyperthermic intraperitoneal chemotherapy (HIPEC) to multivisceral resection/peritonectomy. Other surgical procedures that did not include any of the above procedures were performed in 12.7% of all the patients. Univariate analysis showed some of the factors associated with specific type of surgical management. NET, female gender, non-metastatic disease, any co-morbidity, rural hospital location, and less number of diagnoses were related to appendectomy surgery (Table 3). Apart from co-morbidities and hospital location, these factors remained significant on multivariable analysis (Table 4). Appendectomy was more likely to be performed in patients with NET cancer (31%) as compared to non-NET (17%) (OR: 1.59; 95% CI: 1.23 - 2.07; P < 0.001). Patients with localized disease (HR: 0.64; 0.54 - 0.76; P < 0.001) as well as female patients (HR: 0.65; 0.55 - 0.77; P < 0.001) were more likely to have appendectomy. However, race, age of the patients and insurance status did not show any significant association with the type of surgical management received. Three hundred and twenty-four patients were treated with HIPEC (8.5% of all the cases), mostly with non-NET histology (91% vs. 8%). Majority of these patients had no co-morbid medical illness (60% vs. 39%), and they received their surgical care at hospitals located in urban areas.

**Table 3:** Association of Patient and Facility Characteristics With Choice of Surgical Management

Covariate	Level	Type of surgery		
		Appendectomy (N = 698)	Non-appendectomy procedures including HIPEC (N = 3,101)	P-value
Age (years)	Median (range)	56.5 (12 - 96)	57 (12 - 97)	0.147
Gender	Male (%)	265 (15.55)	1,451 (84.45)	< 0.001
	Female (%)	433 (20.85)	1,643 (79.15)	
Race	White (%)	437 (18.97)	1,882 (81.03)	0.871
	Black (%)	52 (17.91)	234 (82.09)	
	Other (%)	209 (18.08)	985 (81.92)	
Histology	NET (%)	91 (30.96)	200 (69.04)	< 0.001
	Non-NET (%)	607 (17.38)	2,901 (82.62)	
Metastatic cancer	Present (%)	192 (12.39)	1,379 (87.61)	< 0.001
	Not present (%)	506 (22.73)	1,722 (77.27)	
Any comorbidity	≥ 1 (%)	343 (22.28)	1,202 (77.72)	< 0.001
	0 (%)	355 (15.8)	1,899 (84.2)	
Location of hospital	Rural (%)	89 (23.53)	285 (76.47)	0.027
	Urban (%)	609 (17.96)	2,816 (82.04)	
Number of diagnoses	Median (range)	7 (1 - 30)	8 (1 - 31)	< 0.001
In-hospital mortality	Died, N = 96 (%)	6 (6.25)	90 (93.75)	< 0.001
	Did not die, N = 3,703 (%)	692 (18.68)	3,011 (81.32)	
Disposition	Home, N = 2,792 (%)	548 (19.63)	2,244 (80.37)	< 0.001
	Others, N = 1,007 (%)	92 (9.14)	915 (90.86)	

Data are presented as raw number of patients (%) or median (range). NET: neuroendocrine tumor; HIPEC: hyperthermic intraperitoneal chemotherapy; SD: standard deviation.

**Table 4:** Variables Identified Through Predictor Modeling to Have Significant Association With Choice of Appendectomy

Covariate	Level	Type of surgery appendectomy	
		Odds ratio (95% CI)	P-value
Histology	NET	1.59 (1.23 - 2.07)	< 0.001
	Non-NET	-	-
Gender	Male	0.65 (0.55 - 0.77)	< 0.001
	Female	-	-
Metastatic cancer	Present	0.64 (0.54 - 0.76)	< 0.001
	Not present	-	-
Number of diagnoses		0.98 (0.97 - 1.00)	0.060

NET: neuroendocrine tumor; HIPEC: hyperthermic intraperitoneal chemotherapy; SD: standard deviation.

## Discussion

The prognosis and treatment of appendiceal cancers is largely determined byhistology [10]. The 5-year disease-specific survival rates from previous reports range from 93% for NET to 27% for signet ring cell adenocarcinoma, with up to an eight-fold difference in the adjusted hazard ratio within the same cancer stage between patients with differing tumor histologies [11]. Management guidelines for management of NET of the appendix recommend simple appendectomy for tumors less than 2 cm in the absence of high risk features such as mesoappendiceal or vascular invasion, positive or uncertain margins, and mixed histology [6, 7] and right hemicolectomy for tumors longer than 2 cm [12]. Indolent well-differentiated low grade mucinous tumors are managed by surgical resection of primary site and debulking surgery with HIPEC for more advanced disease. In the rare high grade adenocarcinoma or signet ring of the appendix, more extensive surgery with right hemicolectomy and systemic therapy will be required [13]. Therefore, surgery has a central role in the management of appendiceal tumors. In this study, we report on the pattern of inpatient surgical management of appendiceal tumors and the interplay of various clinical and demographic factors such as tumor histology, gender, race and insurance status.

The demographics of the hospitalized patients included in this analysis are comparable to previously reported results [2, 3]. Similar to the results from population-based registry studies, Caucasian females in the sixth decade represented the largest group and cases of non-NET were more common than NET. Patients with NET are younger than patients with non-NET histologies and presented at an earlier stage of disease. Overall the outcome of hospitalized patients with appendiceal tumors was good with relatively low mortality rates and high proportion of patients being discharged home. As expected mortality and shorter hospital stay was seen in patients undergoing appendectomy as compared to other surgeries.

Patients with appendiceal NET and early stage disease were more likely to receive appendectomy. This observation was expected and consistent with current management guideline recommendations. The use of multi-visceral resection and HIPEC at 2.4% was however, much lower than what would be expected in the population with advanced stage non-NET. Numerous studies have established multivisceral resection and HIPEC as the gold standard for pseudomyxoma peritonei secondary to non-NET [14-16], a significant subset considering the 37-38% incidence of mucinous neoplasms in appendiceal cancers [2-4]. The low utilization of debulking surgery and HIPEC was however not associated with patient characteristics, such as race or insurance status. Majority of patients were treated with non-appendectomy surgical procedures. These patients had less co-morbidity and were more likely to be treated at urban centers. The overall usage of HIPEC at 2.4% is however concerning and suggests that patients with advanced stage non-NET of appendix may be receiving suboptimal surgical interventions including HIPEC. While simple appendectomy is employed in the minority of patients both in the rural and urban settings, the majority of cases of non-appendectomy surgical procedures including HIPEC were performed in the urban centers. This may either reflect the larger population of patients residing in urban areas and/or the increased availability of the required expertise at such urban centers compared to the rural areas.

Our analysis is the largest detailed review of the surgical management approach employed for patients with appendiceal cancer in the US. Since procedures such as multivisceral resection and HIPEC are performed in the hospital, the NIS dataset would be sensitive to capture a significant proportion of patients who were treated with this strategy. Nonetheless, our findings have important limitations that should be considered. The retrospective nature of this work limits our ability to full control for potential biases and confounders. Patients included in the dataset cannot be followed longitudinally to determine long-term survival outcomes. Moreover, it is not unlikely for the diagnosis to be unclear at the initial resection and decisions regarding additional surgery to be made after pathologic examination of surgical specimens. Social and personal factors of the patient that influence the choice of a specific surgical intervention cannot be ascertained from this database analysis. Furthermore, we observed a significant number of cases had missing data regarding surgical procedure employed. Finally, the lack of coding for the different non-NET histology precluded analysis of the impact of specific histologic subtypes on the surgical approach.

In conclusion, our study established the utilization pattern of various surgical interventions employed in the management of appendiceal tumors in the US. It is anticipated that these findings will guide future prospective research in this arena especially, studies that will focus on potential barriers to appropriate surgical intervention including HIPEC in patients with advanced stage disease.
